# Supplementary material for: Drug repurposing for aging research using model organisms
Source: Aging Cell. 2017 Jun 16;16(5):1006–15. doi: 10.1111/acel.12626 (PMC5595691; doi:10.1111/acel.12626)
Supplement: Supplementary file 7 — Data S1 Zip‐Archive of all report cards. [file ACEL-16-1006-s007.zip › RC_IM6.pdf]

IM6

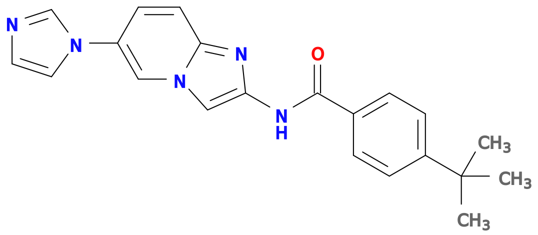

Database identifiers

ChEMBLCompound	CHEMBL1642655

Ranking

	Rank	Score
Drosophila	496/697	0.28
C. elegans	313/591	0.187

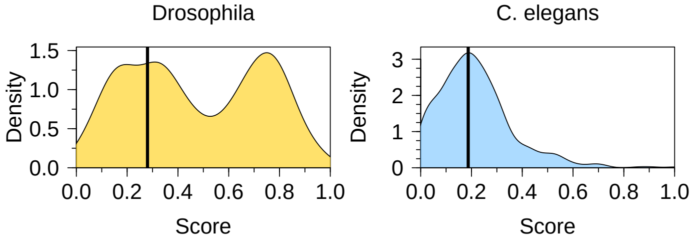

	Ageing implication	Domain conservation	Binding site conservation	Binding affinity	Bioavailability	Lipinski	Promiscuity	Purchasability	Drug approval	**Total**
Drosophila	0.36	0.956	1.0	0.906	(0.9)	0.0	-0.0	0.0	0.0	0.28
C. elegans	0.36	0.922	0.932	0.906	0.67	0.0	-0.0	0.0	0.0	0.187

Names

No synonyms found

Roles

ChEBI entry None has no roles

Status

Approved drug (according to ChEMBL)	No
Number of Rule of 5 violations	0
Binding affinity to original target in log units (RF-Score prediction)	7.26
Burns *C. elegans* bioavailability prediction	4.79

Compound Target Characteristics

Mitogen-activated protein kinase kinase kinase 5

Best gene implication in ageing for this target family came from gene P23561 via mapping the annotation from Ensembl YLR362W via mapping the annotation from EntrezGene 851076 via mapping

the annotation from GenAgeModels 1609 annotated in GenAge release 17.

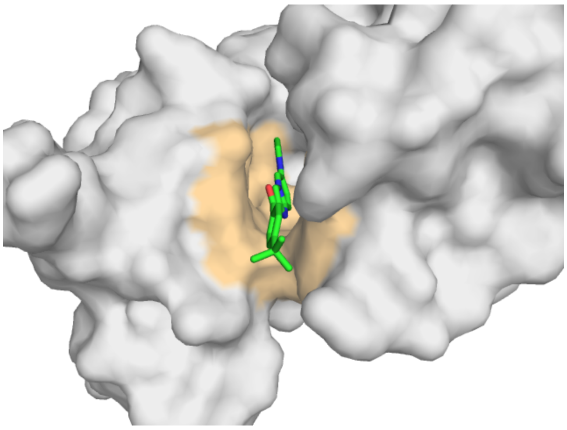

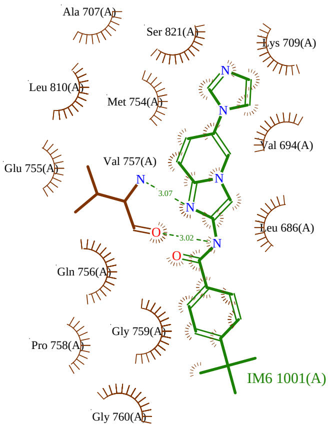

protein	amino acids contacts (binding site)												
PDB:3vw6:chainA:Q99683	L	V	A	K	M	E	Q	V	P	G	G	L	S
sp:Q99683:M3K5_HUMAN	L	V	A	K	M	E	Q	V	P	G	G	L	S
tr:D3ZW27:D3ZW27_RAT	L	V	A	K	M	E	Q	V	P	G	G	L	S
tr:D3ZZH6:D3ZZH6_RAT	L	V	A	K	M	E	Q	V	P	G	G	L	S
tr:E9PWG9:E9PWG9_MOUSE	L	V	A	K	M	E	Q	V	P	G	G	L	S
sp:O35099:M3K5_MOUSE	L	V	A	K	M	E	Q	V	P	G	G	L	S
tr:Q24458:Q24458_DROME	L	V	A	K	M	E	Q	V	P	G	G	L	S
tr:Q7KSB7:Q7KSB7_DROME	L	V	A	K	M	E	Q	V	P	G	G	L	S
tr:Q8I6M5:Q8I6M5_DROME	L	V	A	K	M	E	Q	V	P	G	G	L	S
tr:Q9VDS9:Q9VDS9_DROME	L	V	A	K	M	E	Q	V	P	G	G	L	S
tr:Q4QQD0:Q4QQD0_DROME	L	V	A	K	M	E	Q	V	P	G	G	L	S
tr:B9EQQ2:B9EQQ2_DROME	L	V	A	K	M	E	Q	V	P	G	G	L	S
tr:Q21029:Q21029_CAEEL	L	V	V	K	M	E	H	V	P	G	G	L	S
sp:P23561:STE11_YEAST	I	V	A	K	L	E	Y	V	P	G	G	L	T

	whole protein		domain-based		contact-based	
protein	ident	simil	ident	simil	ident	simil
PDB:3vw6:chainA:Q99683	1.0	1.0	1.0	1.0	1.0	1.0
sp:Q99683:M3K5_HUMAN	1.0	1.0	1.0	1.0	1.0	1.0
tr:D3ZW27:D3ZW27_RAT	0.91	0.96	1.0	1.0	1.0	1.0
tr:D3ZZH6:D3ZZH6_RAT	0.92	0.97	1.0	1.0	1.0	1.0
tr:E9PWG9:E9PWG9_MOUSE	0.94	0.97	1.0	1.0	1.0	1.0
sp:O35099:M3K5_MOUSE	0.94	0.98	1.0	1.0	1.0	1.0
tr:Q24458:Q24458_DROME	0.22	0.38	0.73	0.91	1.0	1.0
tr:Q7KSB7:Q7KSB7_DROME	0.33	0.59	0.73	0.91	1.0	1.0
tr:Q8I6M5:Q8I6M5_DROME	0.36	0.68	0.73	0.91	1.0	1.0
tr:Q9VDS9:Q9VDS9_DROME	0.36	0.68	0.73	0.91	1.0	1.0
tr:Q4QQD0:Q4QQD0_DROME	0.36	0.68	0.72	0.91	1.0	1.0
tr:B9EQQ2:B9EQQ2_DROME	0.36	0.68	0.73	0.91	1.0	1.0
tr:Q21029:Q21029_CAEEL	0.31	0.65	0.62	0.85	0.85	0.93
sp:P23561:STE11_YEAST	0.1	0.28	0.32	0.62	0.69	0.86

Pk92B (FBgn0014006) associated phenotypes

heat sensitive

(Information from FlyBase)

nsy-1 (WBGene00003822) associated phenotypes

aldicarb hypersensitive, anoxia resistant, benzaldehyde chemotaxis variant, butanone chemotaxis variant, cell stress response variant, egg laying defective, egg laying response to food variant, gene expression level reduced, genetic pathway activation variant, lethal, levamisole hypersensitive, loss of asymmetry AWC, odorant adaptation variant, organism high sodium chloride hypersensitive, organism stress response variant, pathogen induced gene expression variant, pathogen susceptibility

increased, pentanedione chemotaxis variant, protein phosphorylation reduced, pyrazine chemotaxis variant, sterile, transgene expression reduced

(Information from WormBase)
